# Nitrate of Silver in Toothache

**Published:** 1858-01

**Authors:** 


*Nitrate of Silver in Toothache.*—The common methods of destroying the nerve in a tooth without extracting it, are the application of nitric or sulphuric acid, or a red-hot wire, but these are very painful expedients.

Some time ago I tried the application of a small piece, about the size of a pin-head, of stick caustic (nitrate of silver) in the hollow of a decayed tooth, from which I was suffering extreme pain. To my great surprise the pain instantly ceased on the caustic touching the nerve, and I had not a return of the toothache for twenty-four hours: when I again applied the caustic, and again got immediate relief, which continued over twenty-four hours. I had occasion to make a third application of the caustic, but since that time, now some months ago, I have not been troubled with the toothache.

This cure will be found ineffectual if gum-boils accompany the toothache, for in that case the decayed part of the tooth is on the outside of it, and, therefore, the application of the caustic to the interior of the tooth can do no good.—DR. J. JOHNSTONE, in *London Lancet.*—*Boston Med. Journal.*

